# Changes to muscle and fascia tissue after eighteen days of ankle immobilization post-ankle sprain injury: an MRI case study

**DOI:** 10.1186/s12891-024-08254-8

**Published:** 2025-01-09

**Authors:** Meeghage Randika Perera, Pan Su, Samantha Holdsworth, Geoffrey Handsfield

**Affiliations:** 1 Auckland Bioengineering Institute, University of Auckland, Auckland, New Zealand; 2 Siemens Medical Solutions, USA, Inc., Malvern, PA USA; 3 Mātai Medical Research Institute, Gisborne, New Zealand; 4 Department of Anatomy & Medical Imaging, Faculty of Medical & Health Sciences, University of Auckland, Auckland, New Zealand; 5 Department of Orthopaedics and Joint Department of Biomedical Engineering, University of North Carolina at Chapel Hill, Chapel Hill, NC United States

**Keywords:** Ankle injuries, Muscle atrophy, Fascia, Magnetic Resonance Imaging (MRI), Muscle hypertrophy, Post-injury recovery

## Abstract

**Background:**

Ankle sprains often result in muscle atrophy and reduced range of motion, which can cause long-term ankle instabilities. Understanding the changes to muscle—such as atrophy—and concomitant changes to deep fascia—which may thicken alongside muscle loss—after ankle sprain injury is important to understanding structural changes about the joint and how they might contribute to longer-term impairments. Here, we employ advanced MRI to investigate skeletal muscle and fascial structural changes during the recovery period of one patient undergoing immobilization after ankle sprains.

**Material and methods:**

In this case study, a participant who suffered an ankle sprain underwent initial MRI scans and, after 21 days (18 of which included immobilization), a follow-up MRI. Techniques used included proton density, 3D stack of spirals, and diffusion tensor imaging to analyse muscle and fascia changes pre- and post-injury.

**Results:**

Results showed muscle atrophy in most shank muscles, with volume loss ranging from no change in the lateral gastrocnemius to 12.11% in the popliteus. Thigh muscles displayed hypertrophy of 6% in the hamstrings, while the quadriceps atrophied by 2.5%. Additionally, fascia thickness increased from 0.94 mm to 1.03 mm. Diffusion tensor imaging indicated that the biceps femoris experienced the most significant changes in physiological cross-sectional area, while the rectus femoris showed minimal change.

**Conclusion:**

The findings highlight the variable responses of muscles and a notable thickening of deep fascia post-injury, underscoring its role in recovery from ankle sprains.

## Introduction

Ankle sprains are the most frequent musculoskeletal injury [1, 2]. Initial ankle immobilization is common but may lead to short term muscle atrophy. Of concern is atrophy compounding the initial injury, complicating recovery and leading to long term ankle instabilities [2]. While muscle atrophy is well-documented, the role of connective tissues such as fascia in recovery remains poorly understood. Fascia, a critical component of the musculoskeletal system, may facilitate force transmission, support structural stability, and contribute to joint function [3]. Understanding how fascia responds to injury and immobilization may shed light on the mechanisms that contribute to longer term impairments like reduced range-of-motion. This in turn is important for informing rehabilitation strategies to prevent long-term chronic instabilities. Research on muscle atrophy after immobilization is thus very important from a clinical perspective. It is common after injury to undergo a reduction of physical activities and bed rest for some amount of time which may lead to muscle atrophy [4, 5]. For example, a common treatment is to apply a cast or wear an Ortho Standard Walker (i.e., Moon Boot) after leg injuries [6], but the ensuing immobilization may could promote muscle atrophy and contribute to joint instabilities in the short to medium term. Previous studies have shown that in the absence of load-bearing, humans experience a 5–20% decrease in knee extensor muscle mass after 3–4 weeks of inactivity [4, 5, 7, 8], and a 12–30% in the cross-sectional area (CSA) of the knee extensor muscles after 4 to 16 weeks of disuse [4, 9].

Measurement of muscle physiological cross section area (PCSA) is used to quantify muscle atrophy after injury [10]. Computation of PCSA is questionable in the absence of fibre orientation data since PCSA is defined as the cross-section orthogonal to the fibre direction [11]. Computation of CSA from only a single axial imaging slice only is limited since axial slices are generally not orthogonal to the muscle’s fibre direction, and this technique also does not capture muscle cross-sections ranging the full length of the muscle. Muscle volume measurements do capture the entire muscle and do not suffer many of the limitations of imaging-based PCSA or CSA measurements. While muscle volume has been shown to correlate with joint torque [12, 13], determination of isometric muscle force generation from muscle volumes remains a challenge, though force is commonly of interest to the biomechanics community. Muscle force-generating capacity depends partly on pennation angles and fascicle lengths, which can remodel from pre- to post-injury, emphasizing the need for noninvasive in-vivo methods to assess these changes [14–16]. Diffusion tensor imaging (DTI) is a useful method for non-invasive quantification of 3D fibre architecture in vivo [17–19]. Implementation of both DTI and muscle volume determination from MRI should enable a relatively robust assessment of the muscular changes occurring during immobilization after ankle sprain.

In addition to changes to muscle that result from ankle sprain injury, there may be alterations to the fascia surrounding the muscle tissue. It has been hypothesised that deep fascia plays a role in transmitting forces between muscles and from muscles to tendons [20]. This may imply that changes to fascia accompany muscle atrophy following injury. This concept notwithstanding, as a peripheral structure surrounding muscle, it seems intuitive that some fascia changes would accompany muscle atrophy or fibre rearrangement following injury. Unfortunately, technical limitations when imaging fast T2* components have thus far prevented in vivo investigation of the fascia pre- and post-injury. Recent advancements have enabled ultrashort echo times (UTE) in MRI to image tissues with very short T2* decay properties [21–23]. UTE for fascia MRI presents an added challenge of needing high spatial resolution due to the thinness of the tissue [24–26]; increased imaging times can maintain signal to noise ratio with increased spatial resolution.

The main aim of this study was to investigate skeletal muscle and fascia during recovery from ankle sprain injury using advanced MRI. Here we use conventional MRI, DTI, and dual echo UTE to assess muscle and fascia architecture in vivo. We collected data from a participant who suffered an ankle sprain shortly after completion of a separate muscle and fascia imaging study. The participant returned for additional data collection 21 days post-injury, with 18 of those days spent in an immobilization boot. We measured muscle volume, muscle PCSA, fascia thickness, muscle fiber pennation angle and fascicle lengths before the injury and after leg immobilization after ankle injury. We hypothesized that the sprain and immobilization would give rise to reduced muscle volumes and PCSAs and increased fascicle thickness. Due to the nature of the unanticipated injury involved in this study, very little information of this kind exists in the literature, making this a unique and interesting case study to probe the changes observed in both the muscles and fascia as a result of injury and ankle immobilization.

## Methods

### Participants

Our participant (female, age: 33 years, mass 54 kg, height 1.69 m) volunteered for MRI scans as part of a study to examine muscles and fascia in healthy subjects. Incidentally, the participant endured an ankle sprain 3 days after initial scan and was prescribed an immobilization boot for 18 days, during which she was allowed limited weightbearing as tolerated. The participant agreed to a follow-up scan 3 days after cessation of immobilization. All scanning involved participant’s right leg and included nearly 80% of the shank, knee, and 70% of the thigh. The participant was informed of the purpose of the study and provided informed consent.

### Magnetic resonance imaging

MRI was conducted on a 3 T MRI scanner (MAGNETOM Skyra, Siemens Healthcare GmbH, Erlangen, Germany). Muscle imaging was conducted using a 2D proton weighted(PDw) turbo spin echo sequence (2D TSE) and diffusion tensor imaging (DTI); fascia imaging involved a dual echo 3D stack-of-spirals research application sequence [27, 28]. Our participant lay feet first supine, with knee nearly fully extended in a comfortable position. A 15-channel knee coil was used to acquire images. The following scan parameters were used for the acquisition.

For PD-weighted imaging: echo time: 38 ms, repetition time: 4780 ms, slice thickness 5 mm, acquisition matrix 512 $\times$ 512, field of view: 250 $\times$ 250, in plane resolution: 0.5 mm $\times$ 0.5 mm. For DTI, we used a spin echo echoplanar imaging sequence with field of view = 249 × 249 mm, acquisition matrix = 92 × 92, slice thickness = 5 mm, in plane resolution 1.3 × 1.3 mm, scan time = 2:30 min, 40 slices, repetition time/echo time = 6000/63 ms, number of signal averages = 1, diffusion directions = 20, b = 500 s/mm2 (reference image with b = 0 s/mm2). For the dual echo UTE sequence: TE1 was 0.05 ms, TE2 was 7.92 ms, repetition time 20 ms, flip angle 5°, slice thickness 3 mm, slice numbers 64, acquisition matrix 512 $\times$ 512, field of view 170 $\times$ 170 mm, in plane resolution 0.3 $\times$ 0.3 mm.

**Fig. 1:**
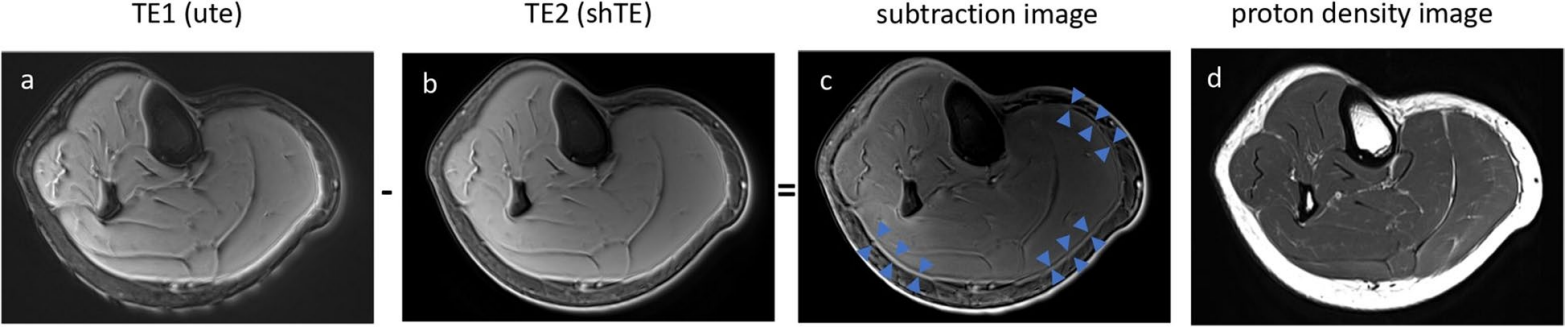
Axial UTE Subtraction and Proton Density Imaging of Lower Leg Post-Injury: Figure (**a**, **b** & **c**) Subtraction of dual echo MR images demonstrates deep fascia tissue with high signal and high contrast (blue arrows in **c**) whereas figure(**d**) shows no signal from deep fascia tissue of the leg

We used a dual-echo Spiral VIBE UTE sequence for fascia imaging as conventional MRI sequences do not use short enough TEs to acquirere signal from fascia. Dual echo imaging is designed to image the same tissue at two distinct echo times—an ultrashort TE and a short TE. Subtracting the short TE from the ultrashort TE images maximizes fascia signal and contrast since there are distinct differences between these signals (Fig. 1) [28].

### Image processing

The same post-processing procedures were used for both imaging time-points unless otherwise stated. Image processing was undertaken using a personal computer (MacBook Pro; Apple, Cupertino, CA, USA). Using ITK-SNAP (http://itksnap.org) the T1 images were used for muscle and bone segmentation in the axial plane and 3D volume rendering, similar to other published studies [29–31]. For noise filtering and smoothing, a standard Wiener and Gaussian filter (3 × 3 kernel size) were used. Since imaging volumes and coverage were not identical pre- and post-injury, image stacks were registered using bony landmarks, specifically the femur for the thigh and the tibia and fibula for the calf, to ensure that the same tissue was studied at both imaging timepoints. The registered stacks were inspected visually to confirm alignment and consistency between pre- and post-scans. We observed minor variations in the positions of subcutaneous blood vessels between imaging timepoints; however, the use of these bony landmarks provides a reliable basis for registration that is less sensitive to movement than soft tissue.

**Fig. 2:**
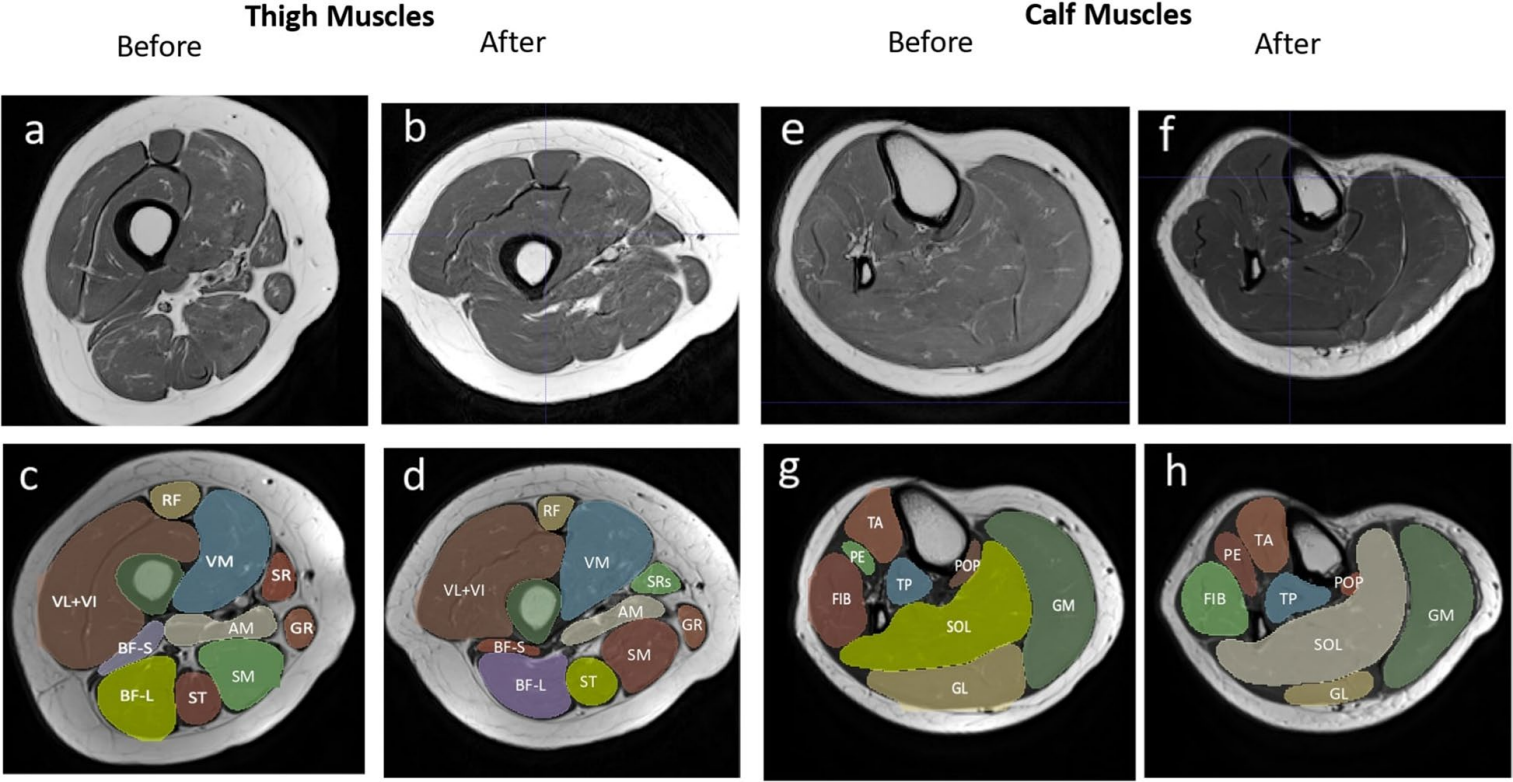
Proton Density MRI of Thigh and Calf Muscles Pre- and Post-Injury with Immobilization: Proton density MRI of thigh (**a**-**d**) and calf (**e**–**h**) region before (**a**, **c**, **e**, **g**) and after (**b**, **d**, **f**, **h**) injury and immobilization. Images were segmented to identify the following muscles: rectus femoris (RF), vastus lateralis + intermedius (VL + VI), vastus medialis (VM), biceps femoris short head (BF-S), biceps femoris long head (BF-L), semimembranosus (SM), semitendinosus (ST), sartorius (SR), gracilis (GR), adductor magnus (AM), tibialis anterior (TA), phalangeal extensors: extensors digitorum and hallucis (PE), peroneals a.k.a. fibulari (FIB), tibialis posterior (TP), popliteus (POP), soleus (SOL), medial gastrocnemius (MG), and lateral gastrocnemius (LG). Note: The pre- and post-injury segmentation images shown are not from the same slice in the limb. Reported quantitative volume analysis provides accurate assessments of changes

After registration, segmentation was undertaken by one trained user who examined each slice of the dataset and referenced a detailed slice-by-slice muscle segmentation atlas used in previous segmentation studies [32] (Fig. 2). Images included roughly 70% of the length of the thigh and 80% of the length of the shank. Muscle and bone (femur and tibia) volumes were calculated by summing the voxel volume of each slice for each muscle or bone.

Dual echo UTE images were used to calculate fascia thickness. In order to reduce bias in measuring the thickness of such a thin tissue, the entire fascia was segmented in the same manner as the muscles.

### Fascia structural measurements

**Fig. 3:**
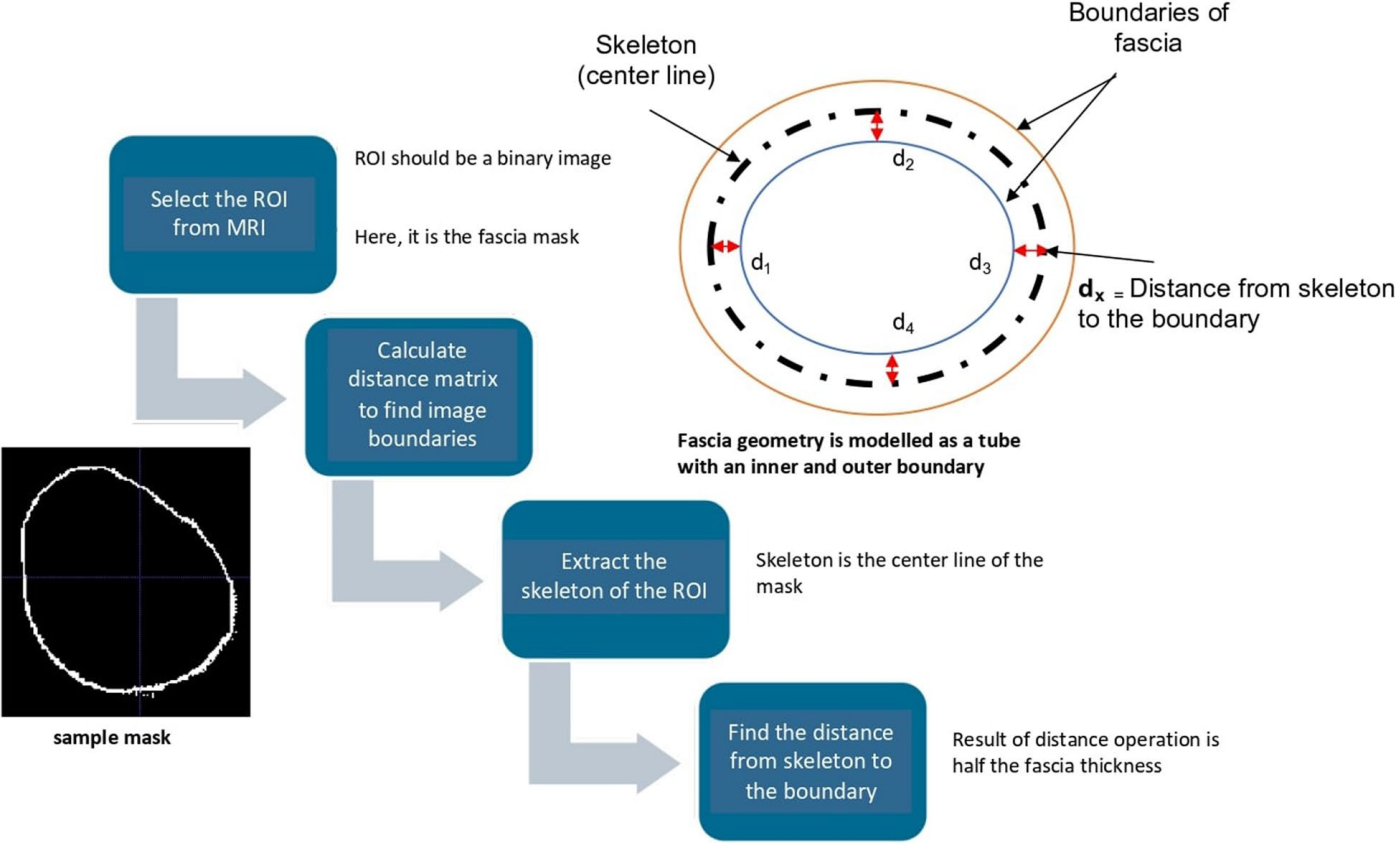
Fascia Thickness Distribution Map Generated by a Computational Algorithm: Fascia thickness distribution map was generated using a computer algorithm. Above diagram illustrates the steps involving in calculating the thickness. The main idea behind the algorithm is to extract the middle line of the geometrical shape of the fascia. Following that, calculating the Euclidean distance from middle line to inner or outer boundary lines would give the thickness measurement for a particular frame. Multiple points of a single frame were selected to calculate the thickness to minimize the error, followed by applying the same procedure for all the other slices of the dataset. Finally, these results which represent spatial thickness distribution of fascia, plotted in a histogram for further analysis

The measurements of interest related to fascia in this study are thickness and thickness variation. Prior work has validated this imaging method for determining fascia thickness in a porcine model [33] and has described the methods and radiological findings in humans [28]. In the present study, a custom algorithm was used to compute the fascia thickness mean and thickness variation (Fig. 3). To this end, the geometrical shape of the fascia was modeled as an irregular tube with an inner and an outer boundary with fascia thickness defined as the distance between these surfaces. We computed Euclidean distance between pixels in binarized images to determine boundaries of the anatomy, then extracting the center using the Matlab function bwskel. Fascia thickness was computed as twice the distance from the center line to the outer boundary. This operation was carried out for multiple points of a single frame to minimize measurement error, and this was implemented across all slices of the dataset, yielding the fascia thickness distribution through the leg.

To check the accuracy of our custom algorithm, we computed the mean thickness using a separate method. Briefly, this method divided the fascia volume by the fascia length and width to determine fascia thickness; here, length was the arclength of the deep fascia curving around the limb and was determined from manual digitization, width refers to the product of the number of slices and slice thickness. The result of this algebraic operation was the mean thickness of the segmented fascia, which was compared to the mean determined from our aforementioned algorithmic approach.

### Diffusion tensor imaging

**Fig. 4:**
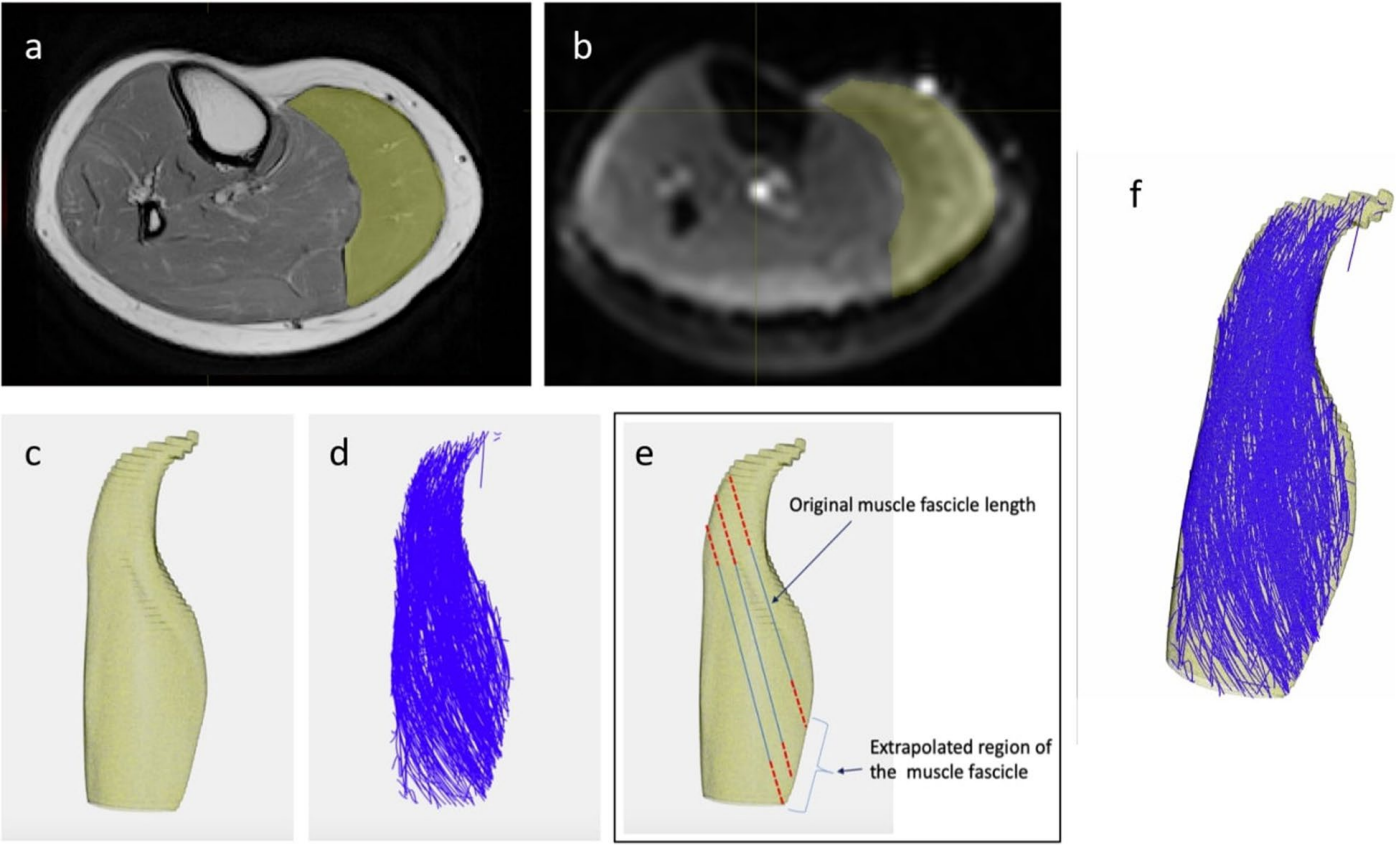
DTI Pipeline for Muscle Architecture Calculation in the Medial Gastrocnemius: Illustration of the DTI pipeline to calculate muscle architecture. **a** axial slice of proton density MRI in the calf region overlaid with a binary mask highlighting the medial gastrocnemius muscle. **b** the same binary mask is here overlaid on the DTI trace image. **c** a surface model of the medial gastrocnemius was created from the binary mask. **d** raw fibre tracts of the medial gastrocnemius derived from DTI. In order to compute muscle fascicle lengths properly, fibre tracts require extrapolation to intersect muscle boundaries (**e**). Extrapolated tracts are overlaid onto the surface of the muscle (**f**) to compute fascicle lengths and pennation angles

DTI was used to track changes to the muscle architecture pre- and post-injury. Preprocessing of DTI data involves denoising and correcting for eddy current distortions, B0-inhomogeneity, and rigid motion correction using algorithms built into FSL (FSL, FMRIB Software Library, Oxford, UK) [34]. In this, the FLIRT rigid registration was implemented in FSL followed by affine transformation to correct small errors between the PD and DTI scans. Diffusion tensors were reconstructed at each voxel to calculate fractional anisotropy (FA) and mean diffusivity (MD) maps using MRtrix (version 3; Brain Research Institute, Melbourne, Australia; http://www.mrtrix.org) [35]. The masks needed for tractography were exported from segmented muscles previously conducted in ITK-SNAP. Tracking parameters were adopted from a previous work [36], which produced 1000 fiber tracts with length ranges from 5 to 200 mm. Since some fibers terminated before reaching the muscle periphery, a custom algorithm was built to extrapolate fiber tracts to muscle boundaries using a polynomial fitting method [37–39] (Fig. 4). Fascicles that were within 30% of the original length were included for further processing.

### Muscle architecture measurements

To compare muscular changes occurring from pre- injury/immobilization to post-injury/immobilization various measurements of muscle architecture were computed from images. For muscle these were volume, fascicle lengths, physiological cross-sectional area (PCSA), and pennation angle. “Fascicle length was defined here as the length between origin of the fascicle and the insertion extrapolated by the algorithm [38]. Pennation angle was the angle between the muscle’s line of action vector and each extrapolated fascicle vector [21]. Muscle physiological cross-sectional area (PCSA) was calculated by dividing the muscle volume by mean fascicle length [21]. The change in femur and tibia volumes were also calculated.

In addition to individual muscle volumes, muscles were grouped into compartments of the calf and thigh. For the calf, the superficial posterior compartment comprised medial gastrocnemius, lateral gastrocnemius and soleus muscles, while the anterior compartment comprised the tibialis anterior, extensor digitorum longus, extensor hallucis longus, and the popliteus. The deep posterior compartment comprised the tibialis posterior, and lateral compartment comprised the peroneals. The thigh’s anatomical groupings were the quadricep/anterior compartment containing the rectus femoris, vastus medialis, vastus lateralis and vastus intermedius; the hamstrings/posterior compartment comprised the biceps femoris short head, biceps femoris long head semitendinosus and semimembranosus; and the medial compartment comprised the adductor muscles and gracilis.

### Statistical tests

All values in this study are presented as mean ± standard error and were statistically analyzed using Matlab 2021A (The Mathworks Inc., Natick, MA, USA) [38]. A general paired ttest was performed following a Kolmogorov–Smirnov test in between pre and post data for all the variables. The alpha level was set to *p* < 0.05. Values that showed significant difference were tested for multiple comparison using Bonferroni post hoc test.

## Results

In the co-registered pre- and post- scans there were volume losses for the bones and most muscles after injury and immobilization. A volume loss was observed in the femur and tibia of 2.22% and 2.47% respectively (Table 1).

**Table 1:** Tibia and Femur Volume Calculations Pre- and Post-Injury with Partial Bone Segmentation: Tibia & femur volume calculations pre- and post- injury. Note that only a portion of each bone could be segmented as the imaging volume only contained approximately 70–80% of each bone

	Pre-injury Volume	Post-injury Volume	Change
Tibia	88.92 cm^3^	86.76 cm^3^	2.47%
Femur	67 cm^3^	65.51 cm^3^	2.22%

Most muscles assessed in this study presented volume loss after injury and immobilization. In the calf, the medial gastrocnemius declined in volume by 2.27%, the soleus declined by 11.2%, the tibialis posterior declined by over 7%, the peroneal muscles declined by 6.11% and the digital extensors declined by over 8%. Only the tibialis anterior and popliteus muscles increased in volume displaying hypertrophy of 3.87% and 12.11% respectively. When grouped as compartments, all muscle compartments in the calf displayed loss of volume in response to injury and immobilization. The superficial posterior compartment (triceps surae) displayed the most significant change of all the compartments with an 8.70% loss of volume. The anterior compartment displayed no change while the deep posterior and lateral compartments show changes of 2.78% and 6.11% respectively.

**Fig. 5:**
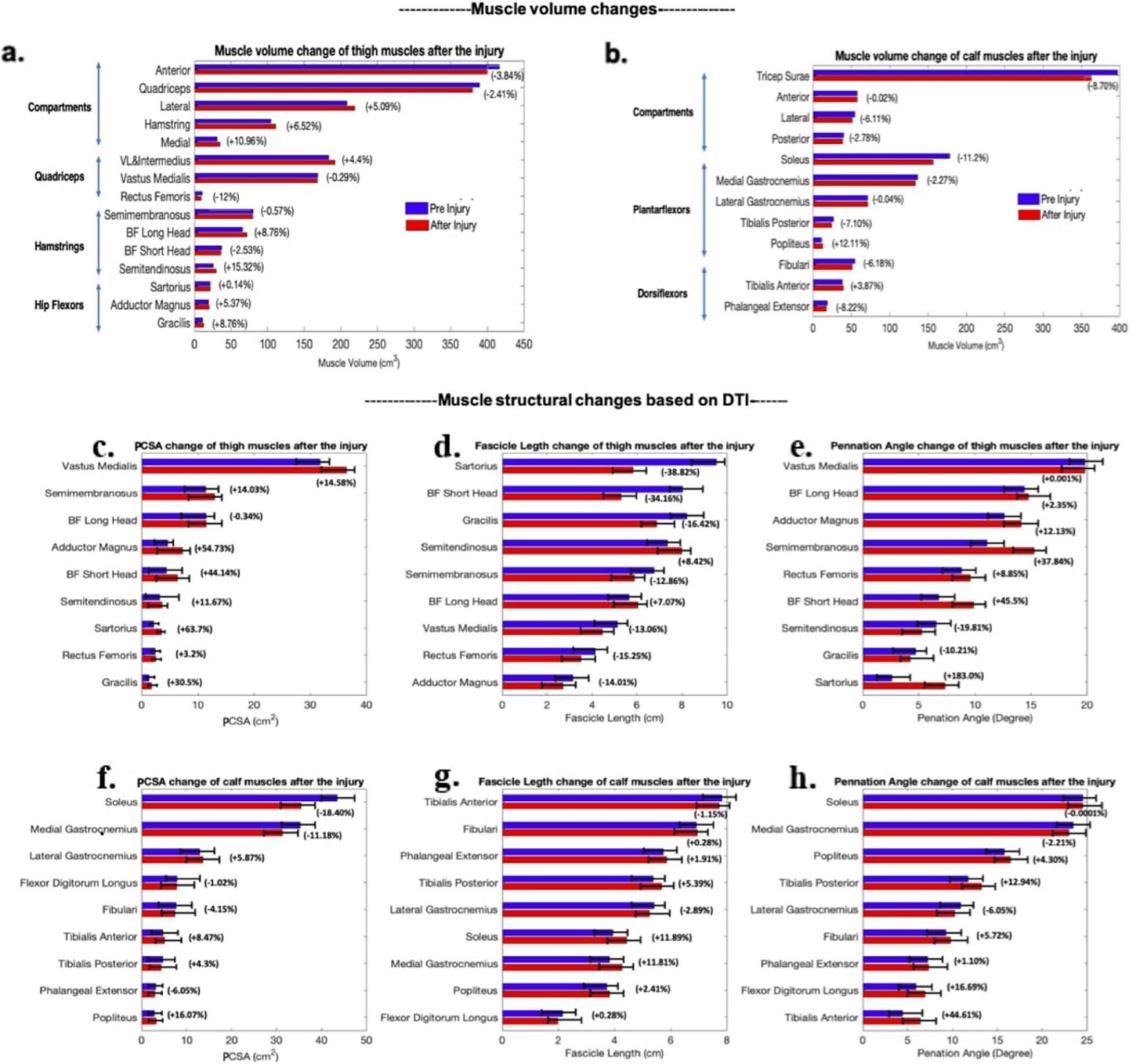
Volume and Architectural Changes in Thigh and Calf Muscles Pre- and Post-Injury with Immobilization: Volume (**a**, **b**) and architecture changes (**c**-**h**) between pre- and post- injury and immobilization for muscles in the thigh and calf. For volume, muscle groups are shown in addition to individual muscles

The thigh muscle volume results were mixed. The semitendinosus displayed the greatest change: a 15% increase in volume. The biceps femoris long head also displayed increases post-injury while the biceps femoris short head decreased in muscle volume by just over 2% (Fig. 5a.). When considered as functional anatomical groupings, the posterior compartment displayed just over 6% increases in volume while the anterior compartment declined in volume by 3.84%. The medial compartment displayed the most pronounced changes with around 11% increases in volume during the recovery phase.

### Pre and post-injury DTI data analysis

In the thigh all muscles increased their PCSA, except for the biceps femoris long head (Fig. 5). For fascicle length, the majority of the muscles showed a decrease or nearly no change post-injury except semitendinosus and biceps femoris long head (Fig. 5). Regarding the pennation angle, there was a general increase across muscles, except the gracilis and semitendinosus muscles that exhibited smaller angles post-injury. Note that not all muscles were included in reports of muscle architecture assessments due to poor DTI data quality for some muscles.

In the calf, six of the nine muscles had reduced PCSA. This included larger muscles such as the soleus and medial gastrocnemius. The fascicle length of most muscles increased, except for the tibialis anterior, flexor digitorum longus, and lateral gastrocnemius. For pennation angle, the soleus showed no change, the lateral and medial gastrocnemius decreased, while the remaining muscles increased following injury and immobilization.

### Fascia thickness measurement

**Fig. 6:**
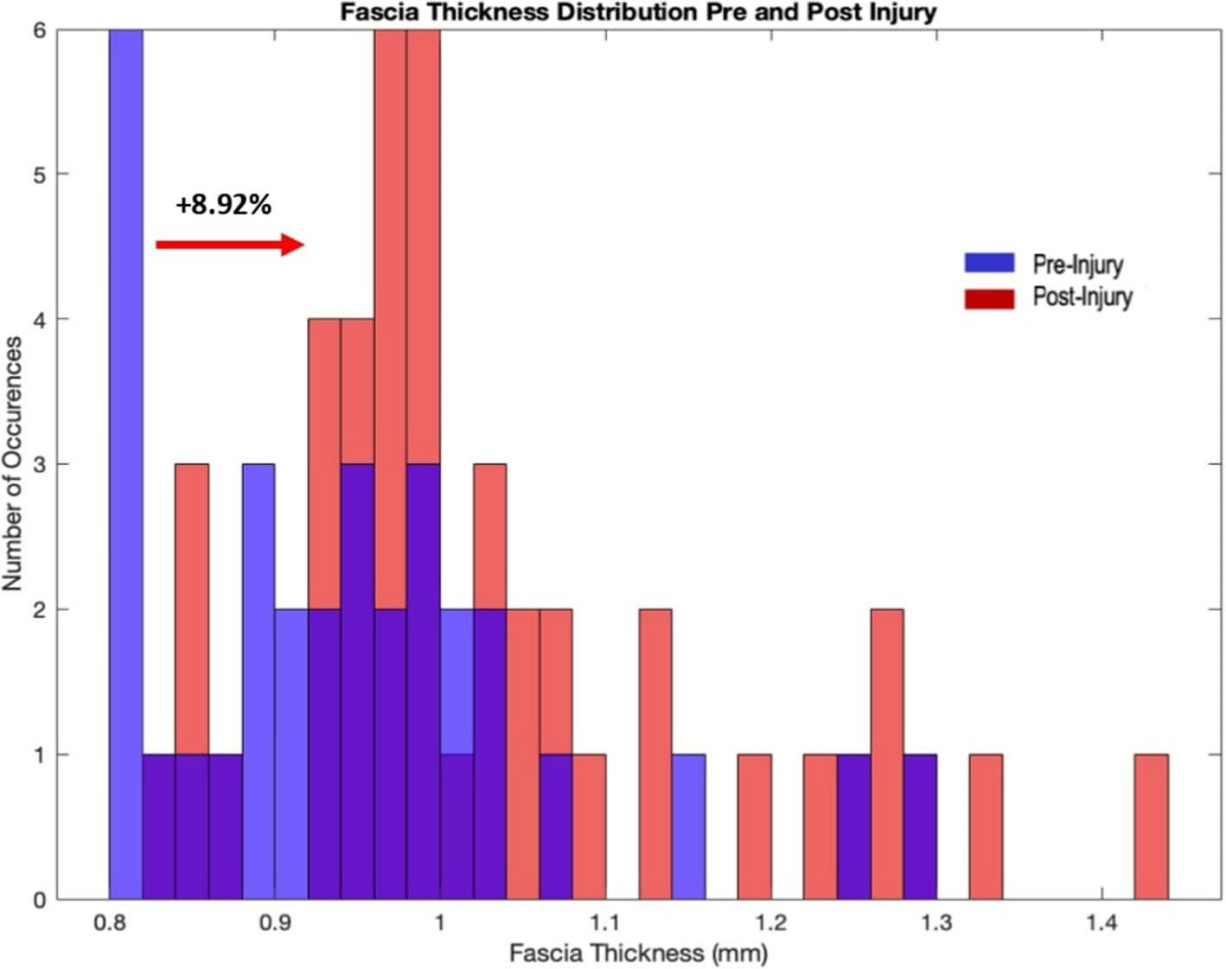
Histogram of Fascia Thickness Distribution in the Lower Limb Pre- and Post-Injury with Immobilization: Histogram of the distribution of fascia thickness in the lower limb pre- and post- injury and immobilization. Post-injury, fascia thickness distribution shifted to the right by 8.92%

Fascia thickness increased as measured by both manual and automated methods. The manual method produced mean thicknesses of 0.94 mm pre-injury, and 1.034 mm post-injury with 8.92% greater thickness post-injury. Consistent with these results, the automated approach indicated a thickening of 10.35% post-injury. Finally, there was an overall thickening of the fascicle distribution of pre- and post- injury (Fig. 6).

## Discussion

This study used MRI to examine the response of lower limb skeletal muscles and deep fascia from before and after immobilization following an ankle sprain ankle injury and immobilization. The major finding of this study is that most calf muscles and some thigh muscles displayed atrophic responses based on volume and fascicle length losses post-injury and immobilization. Furthermore, the deep fascia of the calf appeared to thicken post-injury and immobilization. Although there is general literature support for muscle architectural changes [40–42], this study represents the first MRI-based longitudinal in vivo examination of leg muscles and surrounding connective tissues following injury and immobilization.

Muscle volumes can be obtained from in vivo imaging to robustly assess overall muscle size differences between groups or, in our case, changes from pre- to post- injury and immobilization. Since muscle is generally regarded as isochoric, volume measurements are not sensitive to changes in joint orientation between scans, suggesting that the observed muscle volume changes are related to the injury and immobilization. Indeed, we observed 3–12% volume losses in most calf muscles, along with 3–12% volume gains in the digital flexors, tibialis anterior, and popliteus. Across the thigh muscles there were losses of up to 12%, with some gains of up to 11%. Additionally, we found bone volume losses of 2.22% loss in the femur and 2.47% loss in the tibia. We posit that hypertrophy of thigh muscle volumes may be due to increased demand caused by the extra weight of the moon boot. Alternatively, subtle changes in joint positions may have contributed to these observations; e.g. if the participant was unloading their foot, the absence of weight-bearing would likely result in a flexed knee, which could increase muscular load on the hamstrings. Both mechanisms may have contributed to the observed changes in muscle volume, while the hypertrophy of digital flexor hypertrophy may may have been due to increased use of those muscles while in the moon boot. The loss of muscle volume for most calf muscles is to be expected since the moon boot immobilized many degrees of freedom around the ankle, e.g. plantarflexion, and we would then expect atrophy of underutilized muscles. Bone loss is likely due to decreased loads due to ground reaction forces incident on the bones when the moon boot was in place [43–46].

Our approach to examine structure and thickness of the crural fascia is consistent with literature. Typically, this fascia consists of two to three layers of parallel fiber bundles oriented in multiple directions, separated by thin layers of loose connective tissue. Reported values indicate a total fascia thickness of 924 ± 220 µm, with each layer averaging around 277.6 ± 86.1 µm [47]. This is consistent with our measures derived from dual echo UTE MRI, which showed a healthy fascia thickness of 950 µm. This also aligns with the findings of Pavan et al. [48], further validating both our method and the consistency of crural fascia structure across studies.

Using dual echo UTE MRI, we observed a thickening of the crural fascia, ranging from 8–11%, following injury and immobilization. Fascia thickening around musculoskeletal tissues may be an adaptation to injury and immobilization, as suggested in the literature, where epimysium or fascial thickening has been linked to immobilization, aging [49], and impaired flexibility [50]. Consistent with previous research [47, 51], we found variation of fascia thickness spatially throughout the leg that may be explained by differences in local mechanical loading and force transmission. For example, deep fascia is typically thicker near the ankle to possibly enhance joint stability and proprioception [49], while a decrease in its thickness is associated with recurrent ankle sprains in basketball players [51].

We examined three parameters of muscle architecture: PCSA, pennation angle and fascicle length pre- and post-injury. Diffusion tensor imaging was used to determine muscle fibre directions, which were then used to estimate metrics of muscle architecture. DTI is a powerful tool to detect and quantify changes to muscle fibre structures due to pathology, injury or other reasons [52–58]. Several DTI-based studies have quantified muscle architecture in adult lower limb muscles [19, 38, 39, 59] while others have examined the response to acute or subclinical injury in the same region [60, 61]. These previous studies evaluated muscle fascicle diffusion using eigenvalues, mean FA, and the apparent diffusion coefficient. This current study is, to our knowledge, the first to assess post-injury structural changes in leg muscles using architectural metrics such as pennation angle, fascicle lengths, and PCSA. Among previously published studies of healthy legs using DTI, most investigated the tricep surae muscle group. In this study, we examined seventeen muscles in the thigh and calf, omitting some DTI results—e.g. sartorius, vastus lateralis, and vastus intermedius— as those data were too noisy to generate trustworthy results. When comparing the DTI results of the present study to existing literature, several discrepancies are evident. For example, the previously reported mean fascicle length and PA of MG of healthy adults ranged from 4–5 cm and 10–14° respectively [38, 62]. These previously reported measurements are slightly shorter than the measurement found in the present study. This could be due to several reasons, including ankle and knee positions, and level of muscle contraction [63, 64]. Furthermore, the definition of pennation angle can vary when handling 3D muscle fibre tract data. In our case it was defined as the angle between the muscle’s long axis and the fascicles of the muscle, computed as the arccosine of the dot product of unit vectors. In some studies it is defined as the angle between fascicles and the aponeurosis [18, 65]. One consideration to be made in the present study is the magnitude (40–50%) of length changes observed in some muscles between pre- and post- assessments from the DTI data. It is possible that the muscle positioning during participant setup was at different ends of the range between the pre- and post- acquisition. In our study, we ensured consistent participant positioning and identical joint angles across pre- and post-scan assessments to make sure any observed differences are not attributable to variations in joint angles but rather to other factors. One previous study compared cadaver measurements to in vivo ultrasound measurements of fascicle lengths and pennation angles, indicating large ranges of measurements between relaxed and contracted conditions [66]. Moreover, small muscles have a smaller margin for error, which gives rise to small measurement errors representing a large percent difference for these muscles. Prior repeatability tests have shown mean changes of 3.9% between first and repeated segmentations [38]. In this experiment, the mean difference (MD) for intra-rater assessments was no greater than 1.1 cm^3^ for muscle, with minimal detectable changes (MDCs) below 1.39 cm^3^. For bone, the MD was approximately 0.25 cm^3^, with MDCs under 1.66 cm^3^. For further context, a 2% volume change corresponds to approximately 2.1cm^3^ for muscle and 2.3cm^3^ for bone, demonstrating the method’s ability to reliably detect such changes.

Prior studies have investigated the response of skeletal muscles to immobilization of the lower limb. While planning an injury is not possible, in previous studies participants underwent deliberate immobilization of a single leg for a period of days or weeks with comparisons then being made of muscles between the two legs. Studies have shown that 5–20% decreases in CSA of knee extensor muscles resulted from disuse on the order of weeks [4, 5, 67, 68]. In our study knee extensor muscles showed a 2.55% average muscle loss after 18 days of immobilization. Schulze et.al [8] show that both thigh and calf muscle CSA decreased by 7% after 21 days of unloading period which is consistent with our results. One of the highlights of calf readings is that atrophy response of soleus is much greater than of medial or lateral gastrocnemius, in agreement with several studies [69, 70]. It is thought that the reason for this is that the soleus has more slow twitch fibers, which respond more to unloading compared to the faster twitch fibres of the gastrocnemius [71]. We observed a consistent pattern of increased PCSAs, shorter fascicle lengths, and higher pennation angles in thigh muscles. This general hypertrophy could be attributed to altered loading patterns resulting from the presence of the immobilization boot. Rehabilitation exercises may mitigate injury-induced changes to fascicle length; stretching exercises, for instance, may mitigate the loss of serial sarcomeres due to immobilization, potentially preserving muscle length and range of motion [72]. Disuse and immobilization are likely causing of general muscle atrophy and other changes observed in the calf, while increases in pennation angle and hypertrophy in the thigh may result from alterations in the biomechanics caused by injury and the presence of the immobilization boot.

## Conclusion

Using advanced imaging, we investigated skeletal muscle and connective tissue changes in vivo for pre- and post-injury scenarios of one participant. As expected, many muscles post-injury show atrophic responses while several displayed increases in volume, especially muscles of the thigh that may have hypertrophied because of the added weight of the immobilization boot. Concomitant with muscle atrophy, we observed fascia thickening throughout the limb post-injury. We posit that this may be an adaptive response of this collagenous tissue to injury and immobilization, serving to stabilize the limb and compensating for the space lost due to muscle atrophy. Injury prompts the coordinated migration of fascial fibroblasts, which play a critical role in scar formation and tissue remodeling, demonstrating links between fascia structure and the healing process [73]. Observations of increased collagen content in injured limbs supports the idea that fascia thickening may serve to preserve structural integrity [74]. Future research is warranted to further probe these concepts. Although the nature of the incidental injury in this study limits our sample size to one, this study provides a glimpse at changes occurring during the recovery phase from injury and supports the notion of early rehabilitation work after injury.

## Data Availability

The datasets used and/or analysed during the current study available from the corresponding author on reasonable request.
